# Research on Path Planning Model Based on Short-Term Traffic Flow Prediction in Intelligent Transportation System

**DOI:** 10.3390/s18124275

**Published:** 2018-12-05

**Authors:** Dongjie Zhu, Haiwen Du, Yundong Sun, Ning Cao

**Affiliations:** 1 School of Computer Science and Technology, Harbin Institute of Technology, Weihai 264209, China; 17s030112@stu.hit.edu.cn (H.D.); 18s030126@stu.hit.edu.cn (Y.S.); 2 College of Information Engineering, Qingdao Binhai University, Qingdao 266555, China

**Keywords:** path planning, short-term traffic flow prediction, intelligent transportation system, A-Dijkstra

## Abstract

Vehicle driving path planning is an important information service in intelligent transportation systems. As an important basis for path planning optimization, the travel time prediction method has attracted much attention. However, traffic flow has features of high nonlinearity, time-varying, and uncertainty, which makes it hard for prediction method with single feature to meet the accuracy demand of intelligent transportation system in big data environment. In this paper, the historical vehicle Global Positioning System (GPS) information data is used to establish the traffic prediction model. Firstly, the Clustering in QUEst (CLIQUE)-based clustering algorithm V-CLIQUE is proposed to analyze the historical vehicle GPS data. Secondly, an artificial neural network (ANN)-based prediction model is proposed. Finally, the ANN-based weighted shortest path algorithm, A-Dijkstra, is proposed. We used mean absolute percentage error (MAPE) to evaluate the predictive model and compare it with the predicted results of Average and support regression vector (SRV). Experiments show that the improved ANN path planning model we proposed can accurately predict real-time traffic status at the given location. It has less relative error and saves time for users’ travel while saving social resources.

## 1. Introduction

The development of intelligent transportation system (ITS) requires a high degree of carrying capacity as a guarantee [1,2,3]. Because of their high flexibility and capacity, vehicles are the mainstream means of transportation. Ensuring traffic efficiency will have an important impact on the operation of the city [4]. However, with the continuous increase of vehicle ownership, the insufficient carrying capacity of urban roads has hindered the traffic efficiency of vehicles [5]. Furthermore, the complicated road structure and traffic conditions have caused the load of urban roads to become unbalanced [6]. Therefore, an efficient and accurate path planning method is needed for vehicles to improve the efficiency of urban traffic.

Taxi trajectories [7] is an effective feature of urban road conditions and is widely used in optimizing traffic networks, relieving traffic congestion, and improving the effective development of traffics [8,9]. Almost all taxis in a city are loaded with GPS chips that transmit position information to the service center periodically to provide scheduling, providing call services for management and supervision. The massive amount of GPS data forms large-scale data of taxi mobile trajectories [10]. This massive collection of historical data makes it possible for the transport system to solve various practical problems. In a networked environment, traffic flow and density can be determined by information exchanged between the vehicle and the infrastructure [11]. By using a short-term prediction model, it is possible to estimate travel time according to historical data of vehicle movement, travel time, and vehicle traffic density. However, due to the large amount of data and the uncertainty of the vehicles travel state, traditional travel time prediction model cannot provide a real-time, reliable, and accurate prediction results. So far, there are nearly 30 kinds of prediction methods used in various fields. Some prediction models achieved good results in the long-term prediction application. However, short-term traffic flow has a highly nonlinear, time-varying, and uncertainty characteristics. This means the single feature prediction method cannot meet the prediction accuracy requirements of ITS.

**Figure 1:**
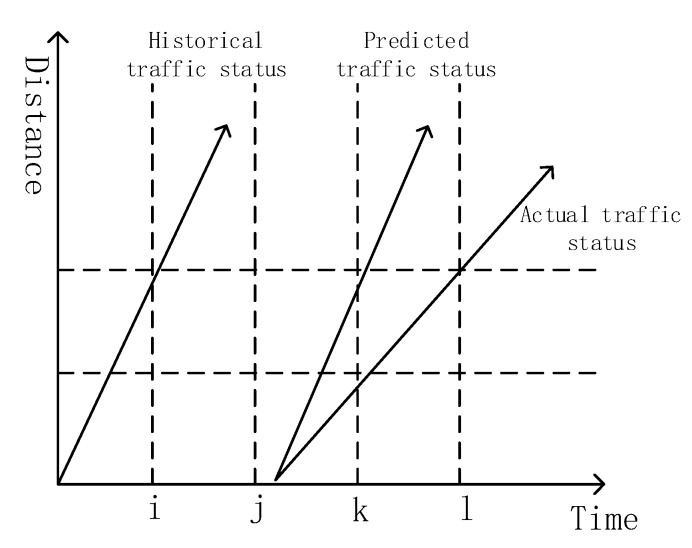
Schematic traffic flow predicting model.

Depending on different methods for predicting short-term traffic flow status, there are four types of prediction models nowadays, including econometric models, neural network model, nonlinear system models, and new emerging technologies, such as dynamic traffic assignment models, data fusion, projection pursuit, data mining, simulation models, etc. A schematic diagram of the general traffic flow predicting model is shown in Figure 1 [12].

The most widely used models are the historical data-based model [13], time series model [14], regression model [15], Kalman filter model [16], and machine learning model [17]. Although the above methods have been proven to work well in many scenarios, there is no effective traffic state prediction model which can feedback the real-time traffic. The recently-developed hybrid prediction model, for example, combines statistical and neural networks [18], a combination of time series and Kalman filter [19]. It has attracted lots of researchers’ attention. Its hybrid composition makes up for the shortcomings of single feature prediction models to a certain extent, but it still cannot completely solve the problem of prediction.

The existing data-associated traffic simulation model and data-driven model classify the above prediction models as a univariate prediction model or a multivariate prediction model [20]. The modern research focus is a multivariate prediction model. Support vector regression (SVR) and artificial neural network (ANN) models are representative of them. ANN and support vector machine (SVM) are classical machine learning methods which have the basic characteristics of the learning method [21]. The main idea of SVM can be summarized as two points: (1) it is the analysis of linear separable case for linearly inseparable samples, SVM uses a nonlinear mapping algorithm to transform linearly inseparable low-dimensional inputs into linearly separable high-dimensional feature spaces. It makes it possible to linearly analyze the nonlinear characteristics of samples using a linear algorithm in high-dimensional feature space. (2) SVM constructs the optimal segmentation hyperplane in the feature space based on the structural risk minimization theory, so that the learner is globally optimized, and the expected risk of the entire sample space satisfies a certain upper bound. ANN [22] is a mathematical model that imitates the behavioral characteristics of animal neural networks and performs distributed parallel information processing. The neural network achieves the purpose of processing information by adjusting the relationship between a large number of internal nodes. Researchers showed that both ANN and SVR can predict travel time on the basis of traffic volume and density [23].

In this paper, we propose a path planning model based on short-term traffic flow prediction. The trace we select is 12,000 taxis GPS data collected during November 2012 in Beijing. Firstly, we divide the massive vehicle GPS historical data by geographical blocks and time blocks and create a data storage structure and processing method. Secondly, we propose a short-term traffic flow prediction model based on ANN. Lastly, we use the traffic speed as a weight to measure the traffic conditions and designed an improved A-Dijkstra algorithm to calculate the optimal path.

In summary, the main contributions of the paper are:

1. We analyze the vehicle trajectory and propose a V-CLIQUES algorithm to gather vehicle status data into clusters. The algorithm can reduce redundant data effectively.
2. We propose a short-term traffic flow prediction model based on ANN to predict traffic speed accurately.
3. We propose an improved Dijkstra algorithm which can effectively solve the problem of traffic congestion and provide an optimal path for a given start point, destination, and departure time.

The paper is organized as follows. The architecture of path planning model is introduced in general in Section 2. Section 3 details the clustering algorithm V-CLIQUE we proposed. In Section 4, we detail a path planning model. Section 5 demonstrates the experimental results of our proposed model. In Section 6 we discuss our findings, and we conclude our work in Section 7.

## 2. Architecture of Path Planning Model

**Figure 2:**
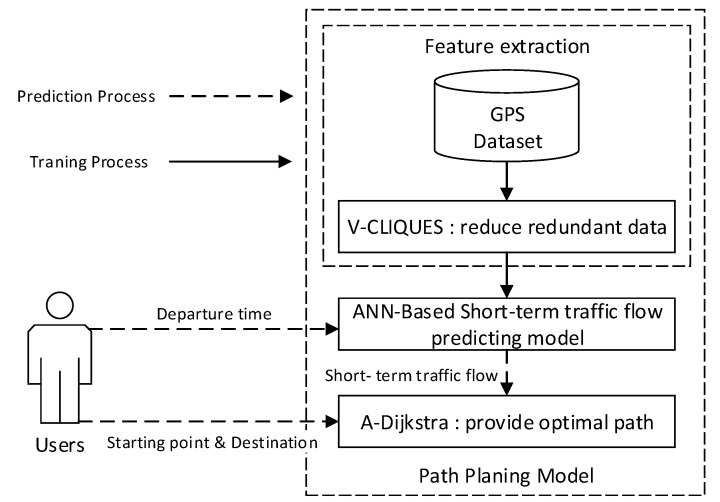
Overall architecture diagram of path planning model.

In this section, the architecture of path planning model is introduced in general. The proposed path planning model is consisted of three parts: V-CLIQUES, ANN-based short-term traffic flow prediction model and A-Dijkstra. We propose V-CLIQUES to extract representative feature in massive GPS data for the purpose of reducing the computational complexity. Moreover, an improved ANN-based model is proposed to predict the short-term traffic flow of the input location, and the traffic condition of the road segment is set as the weight of the path. On this basis, the relative optimal path is provided by the improved Dijkstra algorithm. As a result, the path planning model can plan an optimal path according to a given departure time, starting point, and destination. It can effectively solve the traffic congestion problem. The overall architecture of the path planning model is shown in Figure 2.

## 3. Clustering Algorithm V-CLIQUE

In order to make the prediction results of the model more accurate, the authenticity of the data is critical. We selected 12,000 taxi GPS data collected during November 2012 in Beijing for a total of more than 50 GB of data. We performed statistical analysis on the data and found that if these data are distributed on the map and divided by time directly, about 20% area gathers about 80% of the vehicles.

In this section, we propose a clustering algorithm V-CLIQUE based on the CLIQUE algorithm to analyze the massive taxis GPS data. According to taxis location and density that the dataset provides, V-CLIQUE clusters the duplicate data in one region. Through the processing, simplified and more accurate data of actual speed of the vehicle in the corresponding time zone is obtained, laying a data foundation for the prediction model.

### 3.1. Clustering Algorithm Based on CLIQUE Data

Traditional clustering algorithm has successfully solved the clustering problem of low-dimensional data. However, due to the complexity of the data in practical applications, existing algorithms cannot achieve satisfactory results when dealing with many problems, especially for high-dimensional large data. The main reason is that the traditional clustering method mainly encounters the following two problems when clustering in high-dimensional data sets.

1. There are a large number of unrelated attributes in the high-dimensional data set, so that the possibility of clusters in all dimensions is almost zero;
2. In the high-dimensional space, the data distribution in the lower dimensional space is sparse, and the distance between the data is almost equal. The traditional clustering method is based on distance clustering. Therefore, it is impossible to construct clusters based on distance in high-dimensional space.

### 3.2. V-CLIQUE Algorithm Design

#### 3.2.1. Definition of V-CLIQUE

**Definition** **1.** *Let* A={D1,D2⋯Dn} *be N bounded domains,* S=D1*D2*⋯*Dn *be N-dimensional space.* D1,D2⋯Dn *are dimensions of S.*

*The input to the algorithm is a set of points in an n-dimensional space, set to*$v=\{v_{1},\;v_{2}\cdots v_{n}\}$*where*$v_{i}=\{v_{i1},\;v_{i2}\cdots v_{in}\}$*and the*j-th*component of*$v_{i}$*is*$v_{ij}\in D_{j}$.

*By inputting a parameter*α*, each dimension of the space S can be divided into the same*α*intervals, thereby dividing the entire space into a finite number of disjoint rectangle-like unit, each of which can be described as*$\left\{u_{1},\;u_{2}\cdots u_{n}\right\}$*, where*$u_{i}=\left[l_{i},\;h_{i}\right)$*,*$l_{i}$*is the bottom left corner of the interval*i*and*$h_{i}$*is the top right corner of the interval*i.

$v=\{v_{1},\;v_{2}\cdots v_{n}\}$ *is in an interval* $u=\left\{u_{1},\;u_{2}\cdots u_{n}\right\}$ *, if and only if for each* $u_{i}$ *,* $l_{i}\leq v_{i}<h_{i}$ *is true. We define the selection rate* selectivity(u) *of a unit* u *as follows.*

**Definition** **2.** *We define selectivity is the number of points in the cell to the total number of points in the data space. For the user’s input parameters* β *, data unit u is dense if and only if* selectivity(u)>β *. For any subspace of S, such as subspace* $Sub(S)=D_{t_{1}}*D_{t_{2}}*\cdots *D_{t_{k}}$ *(k < n, and* $t_{i}$ *<* $t_{j}$ *when* i<j *), the same concept can be applied.*

**Definition** **3.** *A cluster can be defined as a connected branch consisting of some connected dense units in a k-dimensional space. The units* $u_{1}$ *,* $u_{2}$ *in the two k dimensions is connected if and only if: (1) the two units have a common face; (2)* $u_{1}$ *,* $u_{2}$ *are all connected to another units* $u_{3}$ *. Two cells* $u_{1}=\left\{R_{t1},\;R_{t2}\cdots R_{tk}\right\}$ *and* $u_{2}=\left\{{R^{\prime }}_{t1},\;{R^{\prime }}_{t2}\cdots {R^{\prime }}_{tk}\right\}$ *have a common face which means that there is a k − 1 dimension (may let this k − 1 dimension be* $t_{1},\;t_{2}\cdots t_{k-1}$ *), there is a* $R_{t1}={R^{\prime }}_{t1}$ *(* j=1, 2⋯k *), and for the* $t_{k}$ *dimension,* $h_{tk}=l_{tk}$ *or* $h_{tk}={l^{\prime }}_{tk}$ *is established.*

#### 3.2.2. Algorithm Specific Steps of V-CLIQUE

The algorithm is based on the CLICQUE algorithm. First, the two-dimensional space (determined by $l_{i}$ and $h_{i}$) distributed by the points is transformed into subunits, and then the number of points of each subunit is counted, that is, the unit density. The selection of the step size S should generally be larger than the average distance of the data items in the data set, and used to mesh the entire space. The density threshold σ is related to the quantity and intensity of the actual data. It will affect the judgment of grid continuity in the clustering process. The initial value can be set according to the actual city size and road density, and dynamically adjusted during the clustering process. The meshing of the obstacles will result in a plurality of consecutive low-density regions. During the meshing process, the initial state of the mesh is unmarked, and after processing, there are two types of state for each mesh: dense mesh and low-density mesh.

### 3.3. Implementation of V-CLIQUE

Step 1: Select the step size parameter S, divide the grid according to the step size and calculate each grid density. The obstacles are meshed and the barrier mesh is marked as a low-density mesh.

Step 2: Traverse all the grids, determine whether the current grids have been marked. If it is, process the next grid, otherwise go to Step 3.

Step 3: If it is a low-density grid, go to Step 2;

Step 4: If the current grid is an unmarked dense grid, assign it to a new cluster marker, create a queue, and place the dense grid into the queue;

Step 5: Determine whether the queue is empty, if empty, then go to Step 2 to process the next grid; otherwise, proceed as follows.

Step 5.1: Take out the grid element of the queue header and check all its adjacent grids that are not processed.

Step 5.2: If the adjacent grid is a dense grid, assign it to the current cluster marker and add it to the queue.

Step 5.3: Go to Step 5.

Step 6: The density connected area is checked, and the same dense grid is marked to form a density-connected area, that is, a target cluster;

Step 7: Modify the cluster tag, perform the next cluster search, and go to Step 2;

Step 8: Traverse the entire data set and mark the data element as the grid cluster tag value.

### 3.4. Implementation of V-CLIQUE

The specific data format is shown in Table 1.

**Figure 3:**
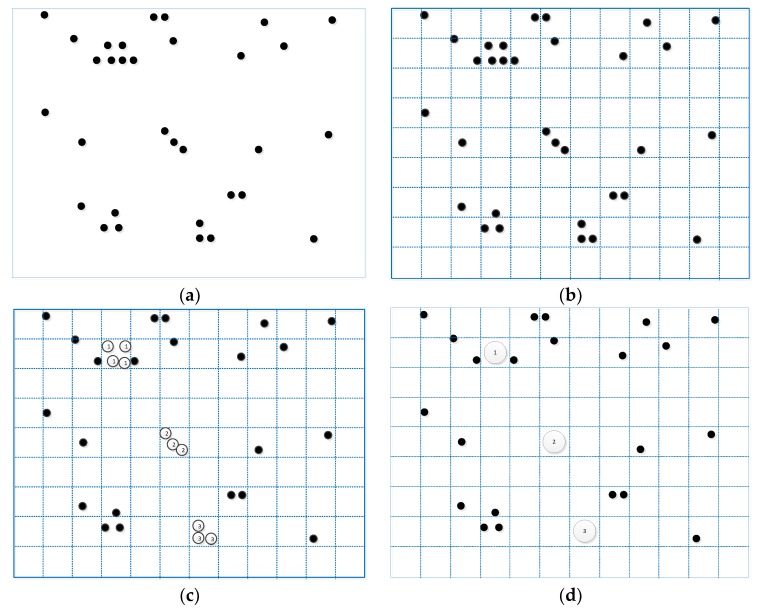
The cluster process is shown in four steps: (**a**) The original sample data; (**b**) The results after dividing the area and setting the density; (**c**) The results of the first clustering; (**d**) The final clustering results.

The cluster process is shown in Figure 3.

The original data is stored in the form of a folder on a daily basis, which contains about 1600 files. The amount of data per day is about 1.6 G. The files processed by clustering are about 450 M per day, and the compression ratio is 27.5%.

## 4. Path Planning Model Based on Dijkstra

The previous section detailed the clustering algorithm V-CLIQUE to preprocess big data and remove some redundant data. For taxis and private cars, the shortest path to the destination is not necessarily the shortest path due to problems such as traffic jams and speed limits. Therefore, the historical traffic condition data of the roads are needed to predict the relatively optimal path.

**Figure 4:**
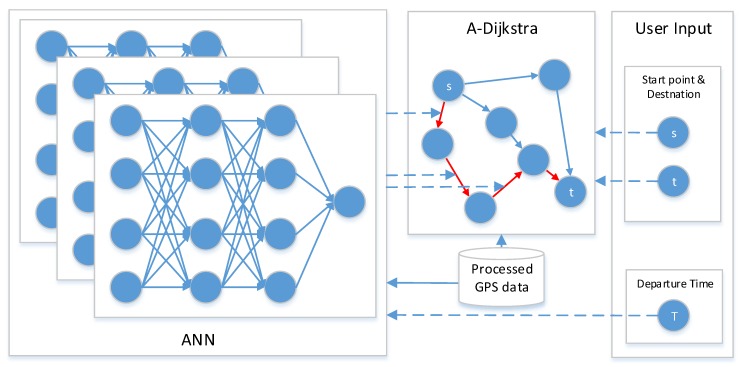
The path planning process of our proposed model. The departure time is inputted to ANN to predict short-term traffic flow. Start point and destination is inputted to A-Dijkstra to find relatively optimal path according to the short-term traffic flow. The red colored arrows in A-Dijkstra are the output of our model, i.e., the relatively optimal path.

Firstly, we propose an ANN-based short-term traffic flow prediction model to predict traffic speed by given time and given roads. The ANN model was trained using GPS data processed by V-CLIQUE. Secondly, a relatively optimal path algorithm A-Dijkstra is proposed to calculate the optimal path according to traffic speed that the ANN model predict. Lastly, for a given start point, destination, and departure time, the optimal path is calculated. The architecture of the model is detailed in Figure 4.

### 4.1. Network Architecture Based on ANN

In the Section 1, we analyzed the advantages and disadvantages of various prediction models. There are many types of ANN model, corresponding to different types of training/learning algorithms. Based on the need of predicting model, we select the back propagation (BP) neural network model, whose learning algorithm steps can be summarized as follows:

Step 1: Initialize the network weights and neurons threshold. The simplest is the random initialization.

Step 2: The forward spread: The input and output of the hidden layer neurons and the output layer neurons are calculated layer by layer according to the formula.

Step 3: The backward spread: Correct the weight and threshold according to the formula until the termination condition is met.

Through the planning function, the algorithm judges the forward propagation result and corrects the network parameters through the backward propagation process to achieve the purpose of supervised learning. Therefore, the traditional BP training process can be summarized into a typical supervised learning process.

**Figure 5:**
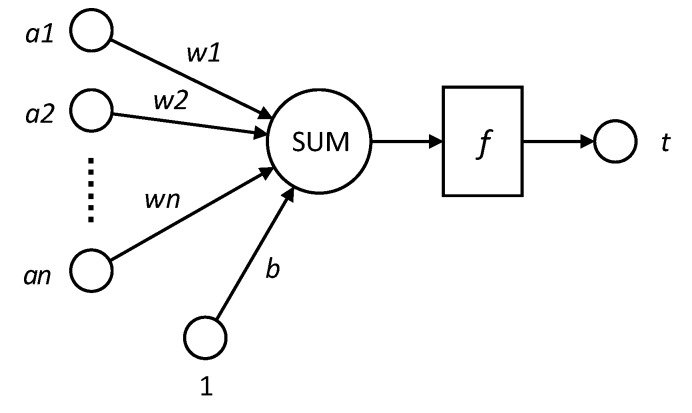
BP neural network model schematic.

The idea of BP can be summarized as follows: Firstly, input vector is presented to the network and propagated forward through the network until reaches the output layer. The output of the network is then compared to the desired output using a loss function. Secondly, error value is calculated for each of the neurons in the output layer. Lastly, the error values are propagated from the output back through the network until each neuron has an associated error value that reflects its contribution to the original output. The BP neural network model topology includes an input layer, a hide layer, and an output layer. The function of a neuron is to obtain the inner product of the input vector and the weight vector and obtain a scalar result through a nonlinear transfer function. The schematic diagram of the specific neural network model is shown in Figure 5.

Among them: *a*1~*an* is input vector for each component; *w*1~*wn* is weights for each neuron synapses; *b* is the bias.

*F* is the transfer function, usually a nonlinear function includes traingd (), tansig (), hardlim (). In this paper, we use hardlim () function. t is the neuron output and $t=f(\vec{W}\vec{A^{\prime }}+b)$. Among them, $\vec{W}$ is the weight vector, $\vec{A}$ is the input vector, $\vec{A^{\prime }}$ is transposition of $\vec{A}$, b is the bias, and f is transfer function.

### 4.2. Training Process

**Figure 6:**
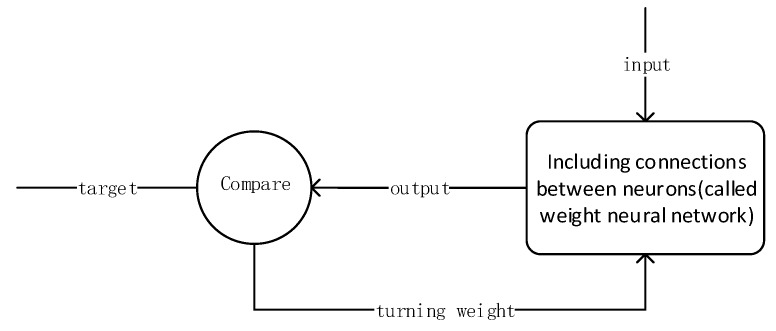
BP neural network node training process.

BP neural network training process nodes shown in Figure 6.

Step 1: Enter vehicle history GPS speed information of road to be trained;

Step 2: Compute connections between neurons using neural network, which means weights;

Step 3: Compare calculated weight value with the actual value;

Step 4: If the error rate is high, then adjust the weights by relearning;

Step 5: Until close to the target, correct rate is between 95–100% range.

### 4.3. A-Dijkstra: Improved Path Planing Model Based on Dijkstra

**Figure 7:**
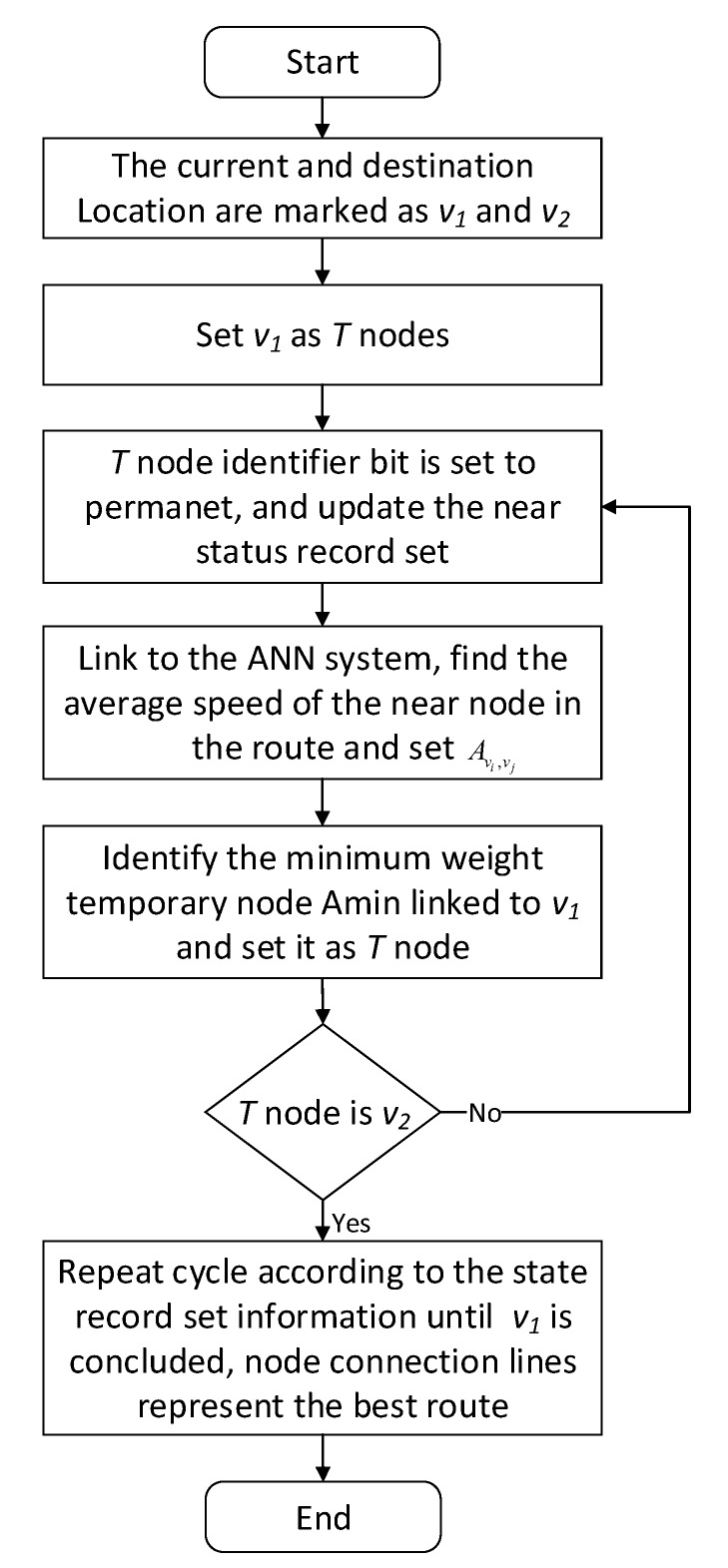
A-Dijkstra algorithm flowchart.

The goal of path planning model is to provide the optimal path for the user’s travel. In this section, we propose an improved ANN-based Dijkstra prediction algorithm, A-Dijkstra. The algorithm uses the length of the road and traffic speed of the urban road at the given time that predicted by short-term traffic flow predicting model. The flowchart of algorithm is shown in Figure 7.

The A-Dijkstra algorithm’s specific steps are as follows:

Step 1: Cut the map into a network diagram includes various sections, and determine the source node $v_{1}$ and the destination node $v_{2}$. Then we create an adjacency matrix F for model, each element in the matrix represents the weight value. For example, $F[v_{i},v_{j}]$ is the weighted traffic speed between nodes $v_{i}$ and $v_{j}$. If there is no road connecting $v_{i}$ and $v_{j}$ directly, their right value to infinity. Set the weight of the road to $1/A_{v_{i},v_{j}}$, where $A_{v_{i},v_{j}}$ is the traffic speed of this road that short-term traffic flow predicting model outputs.

(1) $$F[v_{i},v_{j}]=\{\begin{array}{cc} \frac{1}{A_{v_{i},v_{j}}} & v_{i}\;\mathrm{and}\;v_{j}\;\mathrm{is}\;\mathrm{connected}\;\mathrm{by}\;\mathrm{roads}\\ infinity & other \end{array}$$

Step 2: The model created a set of node status record for each node in the road. This record contains three fields:

Preamble field—indicates the road before the current.

Length field—indicates sum of weight values from the source node to the current sections.

Label field—indicates the status of road. Each road may be in the state of ‘permanent’ or ‘temporary’.

Step 3: Model initialization. Set the length field in the status record set of all nodes to ‘Infinity’ and the label field to ‘Temporary’.

Step 4: Set a node *T*. For example, if you set source node $v_{1}$ as *T*, the model changes $v_{1}$’s label field to ‘permanent’. After a label became ‘permanent’, it will not be changed. *T* node is called a temporary node, in fact, it is merely a proxy.

Step 5: Model update. Update all of the record set which Label field is in the ‘temporary’ status and connected to the *T* node directly.

Step 6: Update *T* node. The model selects the node with the smallest distance from the $v_{1}$ in all ‘temporary’ roads, which is updated to the new *T* node as *T’*.

Step 7: If this node is not $v_{2}$ (the destination node), the model returns to Step 5.

Step 8: If the node is $v_{2}$, then move back to its previous node and extract nodes from state records set, repeat until extract $v_{1}$.

## 5. Prediction Accuracy Assessment

Predicting accuracy is an important indicator to measure the fit of a predictive model. It can describe the degree to which the predicted value produced by the model fits the historical actual value. In this section, we evaluate the proposed path planning model. For path planning problem, there may be multiple relative optimal paths from the starting point to the destination. It is not reasonable to directly compare the path obtained by the model with the path of the actual optimal path. The proposed path planning model is based on the short-term traffic flow prediction model to accurately predict the traffic conditions of urban road sections. Therefore, the predicting accuracy of urban traffic conditions is a guarantee that the model can derive a relatively optimal path.

We compare the predicted traffic conditions with actual traffic conditions of urban road sections. Traffic speed is chosen as an evaluation index. The mean absolute percentage error (MAPE) is selected as the measurement index of prediction accuracy [24]. The absolute value of the average relative error is shown in Equation (2).

(2) $$MAPE=\frac{1}{n}\sum_{i=1}^{n}\left|\frac{y_{i}-{\hat{y}}_{i}}{y_{i}}\right|$$

where $y_{i}$ is actual traffic speed, ${\hat{y}}_{i}$ is the traffic speed that model predicted, and n is total number of road sections. The smaller the value of MAPE, the more accurate the prediction result.

In this paper, we use MATLAB for data processing and simulation. We compared proposed path planning model with the average (Average) and classic SVR-based prediction results. The results are shown in Figure 8, Figure 9, Figure 10, Figure 11, Figure 12, Figure 13, Figure 14 and Figure 15.

**Figure 8:**
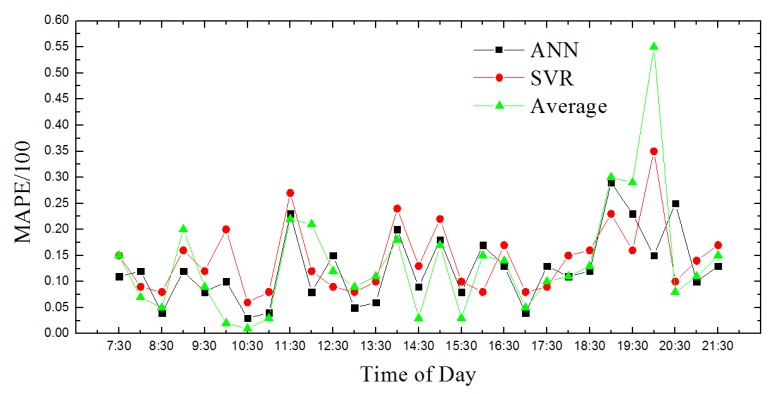
Day’s prediction average absolute percentage error contrast 1.

**Figure 9:**
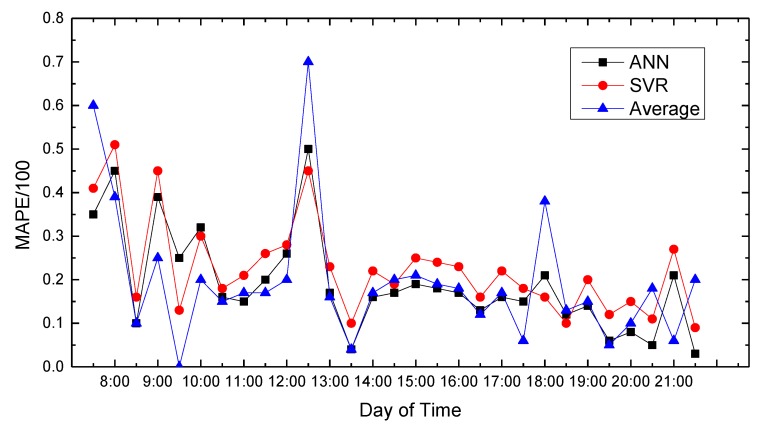
Result of MAPE on Monday.

**Figure 10:**
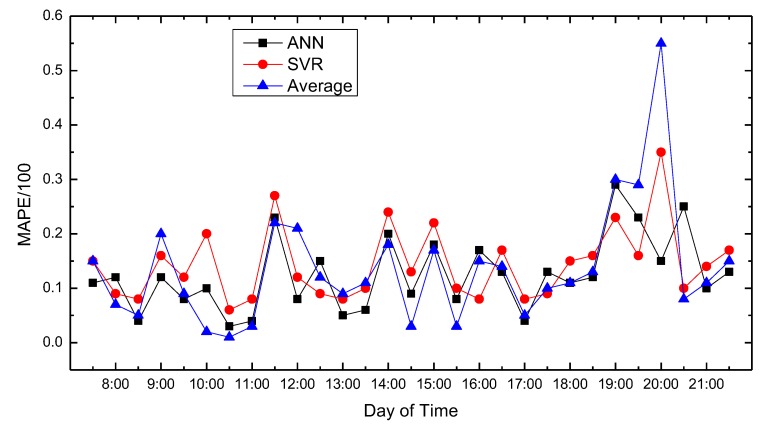
Result of MAPE on Tuesday.

**Figure 11:**
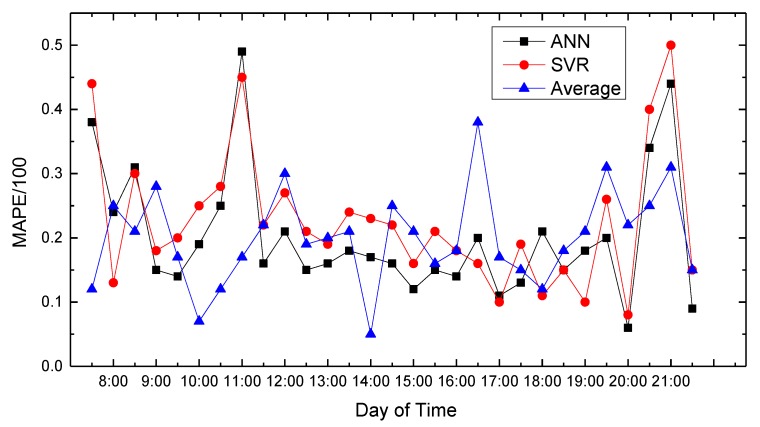
Result of MAPE on Wednesday.

**Figure 12:**
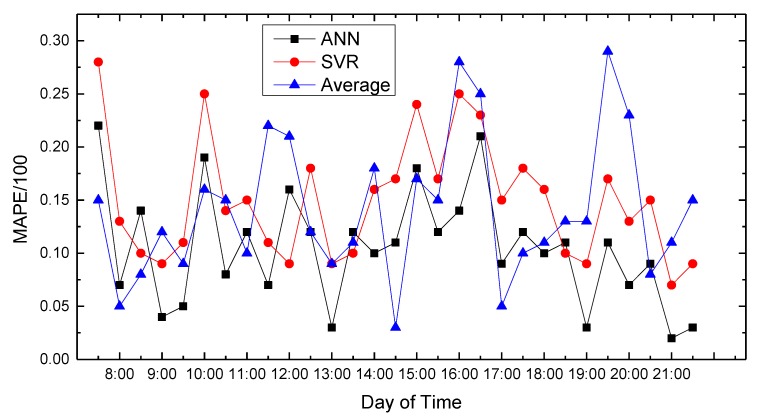
Result of MAPE on Thursday.

We compare the predicted results that the model output with the actual traffic data. For the data of the working day, the experiment found that the average absolute percentage error predicted by the model is similar to the actual data. Figure 8, Figure 9, Figure 10, Figure 11 and Figure 12 show the comparison of the average absolute percentage error of the non-weekend prediction result. It can be seen that the predicted average absolute percentage error of different working days is similar. Experiments show that, on a working day, the improved path planning model can accurately predict the traffic situation. Compared with the average speed based and SVR-based prediction model, the proposed model has less relative error and more accuracy. Therefore, it can provide an optimal path more accurately.

Figure 7 show the average absolute percentage error results for weekend traffic conditions that the model obtained. Analysis showed that because people’s travel time and destination are very different between weekends and weekdays, and the purposes of travel every weekend have great randomness, the predicted average absolute values change greatly, and the prediction accuracy decreases.

## 6. Discussion

The path planning model proposed in this paper can predict urban traffic conditions based on historical traffic conditions and provide relative optimal paths. The main contribution of this model is applying the short-term traffic flow prediction model to the path planning problem under complex urban traffic conditions. The proposed model can be widely applied in urban traffic scheduling, unmanned path decision-making, and other fields.

Since the trace in this research is taxis’ GPS historical data, data still have some errors with the actual road data. Taxis may reduce their speed when it is looking for passengers, and it will take some time for passengers to get on and off. In this paper, we tried our best to find a taxi carrying passengers with historical GPS data as an experimental vehicle node. In addition, the phenomenon of carpooling leads to inaccurate data. It can be predicted if use the private cars’ real data as a training base data, predict results by ANN-based short-term traffic flow predicting model proposed in this paper will be more accurate.

Next, for new vehicle historical GPS data, the model needs to fine-tune the weights according to the new data, so that the prediction model can more accurately predict the vehicle travel time and provide more time-saving paths.

## 7. Conclusions

In this research, we analyze a massive collection of vehicle GPS data and establish a prediction path planning model based on short-term traffic flow. Firstly, the CLIQUE-based clustering algorithm V-CLIQUE is proposed to cluster the historical vehicle GPS data. Secondly, an ANN prediction model is proposed. Finally, the weighted optimal path algorithm A-Dijkstra based on ANN is proposed to achieve the purpose of calculating the most time-saving path so that private cars and taxis can plan their paths according to the model to save time. By performing MAPE evaluation on the prediction model and comparing the prediction results with Average and SRV, the A-Dijkstra model proposed in this paper can predict the real-time traffic situation at the given location more accurately with less relative error. Our extensive evaluations demonstrate that, compared with average and SVR-based path planning methods, the proposed path planning model can find the relatively optimal path more accurately and efficiently.

## Figures and Tables

**Figure 13:**
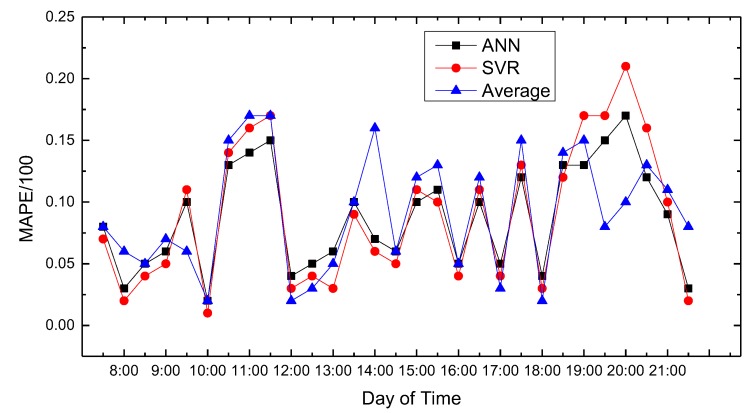
Result of MAPE on Friday.

**Figure 14:**
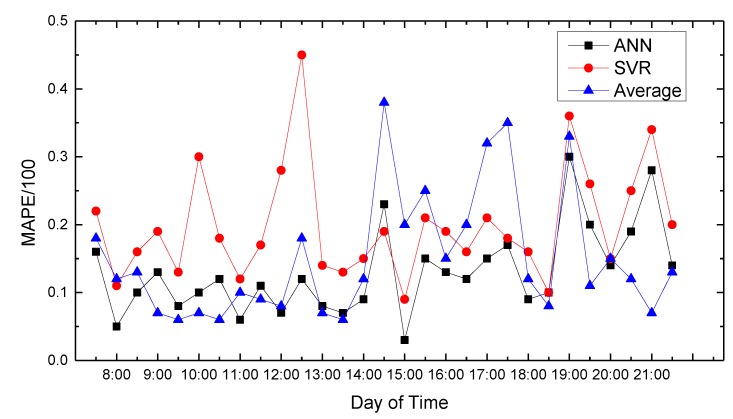
Result of MAPE on Saturday.

**Figure 15:**
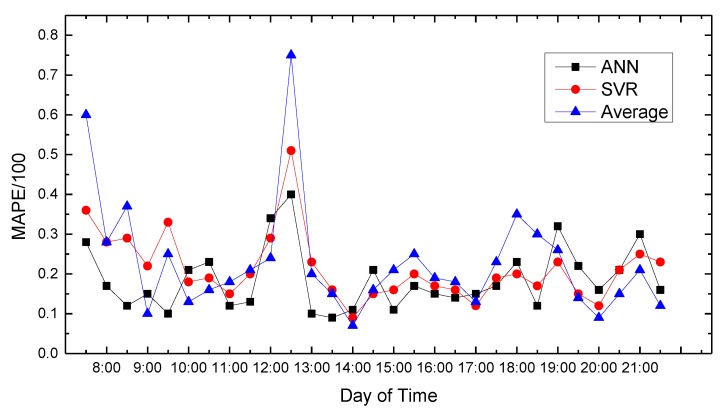
Result of MAPE on Sunday.

**Table 1:** Raw data format and representative significance

Data Item	Data Format and Meaning
Vehicle ID	6 char
Trigger	0 = to vacant, 1 = to occupied, 3 = other
Vehicle status	0 = vacant, 1 = occupied, 2 = parked, 3 = outage, 4 = other
GPS time	Format = yyyymmddhhnnss, China Standard Time
GPS longitude	Format = dd.ddddddd, 0–90° N degrees
GPS latitude	Format = dd.ddddddd, 0–180° E degrees
GPS speed	Format = ddd, 000–255 km/h
GPS direction	Format = ddd, 0–360 degree
GPS status	0 = invalidate, 1 = validate
Ending	newline
